# Conduction system pacing in pediatric and congenital heart disease

**DOI:** 10.3389/fphys.2023.1154629

**Published:** 2023-03-24

**Authors:** Henry Chubb, Douglas Mah, Anne M. Dubin, Jeremy Moore

**Affiliations:** ^1^ Division of Pediatric Cardiology, Department of Pediatrics, Stanford University, Palo Alto, CA, United States; ^2^ Department of Cardiology, Boston Children’s Hospital, Department of Pediatrics, Harvard Medical School, Boston, MA, United States; ^3^ Division of Cardiology, Ahmanson/UCLA Adult Congenital Heart Disease Center, Department of Medicine, University of California Los Angeles Medical Center, Los Angeles, CA, United States; ^4^ Cardiac Arrhythmia Center, David Geffen School of Medicine, University of California, Los Angeles, CA, United States; ^5^ Division of Cardiology, Department of Pediatrics, UCLA Medical Center, Los Angeles, CA, United States

**Keywords:** conduction system pacing, pediatric, congenital heart disease, heart failure, transposition of the great arteries (TGA), left bundle area pacing, His bundle pacing (HBP), pacing induced cardiomyopathy

## Abstract

Conduction system pacing (CSP) has evolved rapidly to become the pacing method of choice for many adults with structurally normal hearts. Studies in this population have repeatedly demonstrated superior hemodynamics and outcomes compared to conventional pacing with the recruitment of the native conduction system. Children and patients with congenital heart disease (CHD) are also likely to benefit from CSP but were excluded from original trials. However, very recent studies have begun to demonstrate the feasibility and efficacy of CSP in these patients, with growing evidence that some outcomes may be superior in comparison to conventional pacing techniques. Concerns regarding the technical challenges and long-term lead parameters of His Bundle Pacing (HBP) have been overcome to many extents with the development of Left Bundle Branch Area Pacing (LBBAP), and both techniques are likely to play an important role in pediatric and CHD pacing in the future. This review aims to assimilate the latest developments in CSP and its application in children and CHD patients.

## 1 Introduction

Over the past 5 years, the advancement of conduction system pacing (CSP) in the management of adult patients with structurally normal heart has been exponential. However, the use of this technology in pediatric and congenital heart disease (CHD) patients has been limited. As is commonly observed in the development of new techniques, fewer patients, younger age at implant and increased patient complexity have inhibited early adoption or inclusion in prospective clinical trials. This review aims to summarize the pertinent and applicable findings in the adult (non-CHD) literature, as well as the limited pediatric and CHD reports of CSP. In this way we aim to provide a platform for the clinician working in the pediatric/CHD field to make an informed decision regarding the merits of CSP in this important patient population.

## 2 Conduction system pacing overview

### 2.1 Anatomy and nomenclature

**FIGURE 1:**
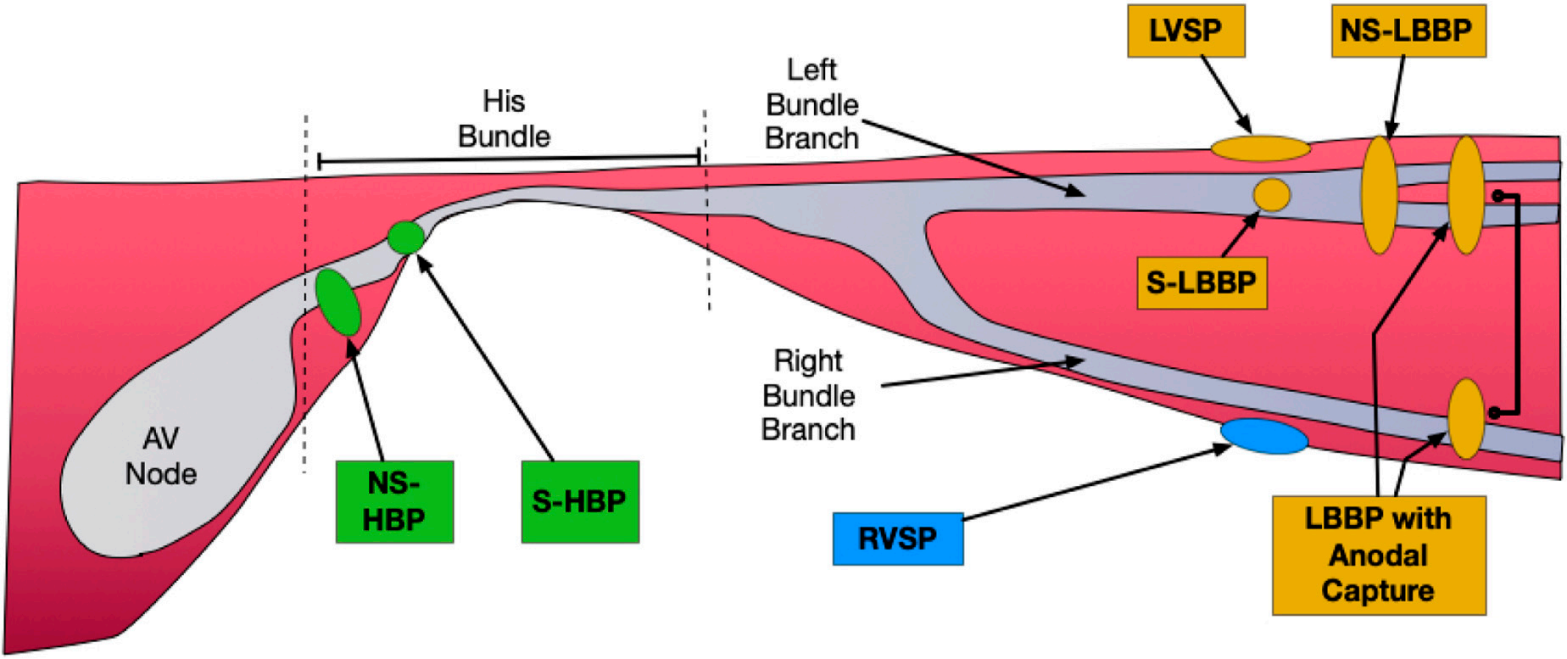
Forms of Conduction System Pacing. His Bundle Pacing (HBP) in green: Selective (without capture of local myocardium: S-HBP) and Non-Selective (with capture of local myocardium: NS-HBP). Forms of Left Bundle Branch Area Pacing (LBBAP) in orange: Selective (S-LBBP) and Non-Selective (NS-LBBP), and additionally LV Septal Pacing (LVSP- capture of the left side of the septum without capture of the LBB), and LBBP with Anodal Capture (anodal ring electrode captures the RBB in addition to LBB capture by tip electrode). RV septal pacing (RVSP) in blue.

There are currently two main forms of CSP: His Bundle Pacing (HBP) and Left Bundle Branch Area Pacing (LBBAP), with multiple subdivisions within each type. (Figure 1). HBP is generally divided into selective (without capture of local myocardium: S-HBP) and Non-Selective (with capture of local myocardium: NS-HBP). LBBAP can include selective (S-LBBP) and non-selective (NS-LBBP) left bundle branch (LBB) pacing, and additionally LV Septal Pacing (LVSP- capture of the left side of the septum without capture of the LBB). In addition, LBBP with anodal capture of the right bundle branch (RBB) is sometimes observed, resulting in LBBAP without RBBB morphology.

### 2.2 Evolution of techniques and evidence in the adult field

HBP was demonstrated as early as 1970, with the first pacing system implant demonstrated in 1992 in canines. (Narula et al., 1970; Karpawich et al., 1992). The first intentional human permanent HBP was described in 2000 by Deshmukh and others, (Deshmukh et al., 2000) and the field has grown rapidly over the last decade. The clinical effectiveness and safety profile of HBP have been demonstrated in numerous prospective studies. (Muthumala and Vijayaraman, 2021). It has been shown that there are significant advantages over conventional RV pacing through utilization of the intrinsic conduction system and subsequent minimization of inter- and intra-ventricular dyssynchrony.

However, there remain two primary concerns that have held back a universal deployment of HBP. The first is the more challenging deployment of a permanent HBP system (rather than RVP), with a long learning curve and persistent 10%–20% HBP system implant failure rate, despite the increasing involvement of device manufacturers in optimization of lead and catheter design. (Zanon et al., 2018). This is particularly relevant in CHD pacemaker implant, where operator volumes are generally much lower, and the anatomy is more complex. The second relates to concerns in long-term lead parameters, with demonstrated significant increases in thresholds, decreases in sensing, and an increased rate of lead dislodgement (4.3% *versus* 0.4% in one large study). (Sharma et al., 2015). In the younger pediatric and CHD population, long-term lead parameters are of paramount importance in order to minimize lifetime procedures and risk.

In this context, the concept of LBBAP has developed rapidly as it has a shorter learning curve, superior medium-term (and potentially long-term) lead parameters and higher rates of successful implant. The technique was first formally described by Huang et al. (2017), and involves the placement of the ventricular pacing lead deep within the basal RV septum with recruitment of either the proximal left bundle or one of its fascicles at the septal myocardium at low output. (Huang et al., 2017). Since those early feasibility studies, the technique has prospered and for many is now the favored approach to CSP in the structurally normal heart: LBBAP deployment is rapidly accelerating ahead of HBP in many centers in advance of formal evidence of superiority. (Padala and Ellenbogen, 2020; Kircanski et al., 2022). Early-to medium-term outcomes of LBBAP have been good, but the novelty of the treatment means that formal evidence of superiority in many measures of efficacy are currently lacking. (Liu et al., 2021).

### 2.3 Indications for conduction system pacing

Pediatric and CHD patients are at risk of cardiomyopathy either secondary to long-term pacing or cardiac dyssynchrony, secondary to existing conventional (single-site, non-CSP) pacing or inherent conduction disease. Traditional biventricular pacing (CRT) has been used with some success in these scenarios (Chubb et al., 2020), but there is growing interest in CSP both to treat and prevent this issue.

#### 2.3.1 Pacing induced cardiomyopathy

In the structurally normal heart, it is well-established that higher conventional right ventricular pacing (RVP) ventricular pacing percentage (Vp) is associated with increased risk of deterioration in systemic ventricular function, heart failure (HF) and death. (Sweeney et al., 2003). Animal studies have also demonstrated that the cardiac dyssynchrony associated with conventional RVP may induce pathological remodeling related to regional differences in wall stress and myocardial work, with subsequent abnormalities in intracellular and extracellular regulation. (Khurshid and Frankel, 2021).

However, the majority of paced patients are subjected to decades of pacing-induced dyssynchrony and never develop pacing induced cardiomyopathy (PICM). Estimates of incidence depend in part upon duration of follow-up (and definition of PICM), but are generally around 10%–20%. One of the larger studies included 823 consecutive patients with RVP and normal baseline function: 101 (12.3%) developed PICM over a follow-up of 4.3 ± 3.9 years. It has been shown that there are factors that pre-dispose to PICM such as male sex, wider QRS duration (native and paced) and higher Vp percentage, but these predictors are relatively weak and many of the determinants of the vulnerability to PICM remain unidentified.

Lead positioning has been studied as a potential risk factor for later development of PICM. Epicardial LV apical lead placement has been shown to decrease the likelihood of developing PICM. (Janoušek et al., 2013). Initial studies suggested that RV septal leads are preferrable over RV apical leads. However randomized trials in non-CHD adults and retrospective studies in children have not demonstrated any superiority in long term outcomes with RV septal lead over RV apical lead. (Janoušek et al., 2013; Kaye et al., 2015). There are few studies specifically looking at CSP and the development of PICM, but early results appear promising. (Gordon et al., 2022). Key indices, such as EF and dyssynchrony measures, trend to improved values with CSP over RV septal leads, but a true mortality or morbidity benefit remains to be established. (Kronborg et al., 2014).

#### 2.3.2 CSP for cardiac resynchronization therapy

CSP is increasingly recognized as a potential alternative modality of cardiac resynchronization therapy (CRT) in the adult population, with demonstration of resynchronized ventricular activation. Initially CSP was described as a bail-out strategy when conventional multisite CRT could not be achieved or failed, but CSP may also be employed as a primary CRT strategy. (Sharma and Vijayaraman, 2021). Furthermore, CSP may also be combined with more conventional, multisite CRT systems, such as His-Optimized CRT (HOT-CRT, with HBP and conventional LV epicardial lead) or LBB- Optimized CRT (LOT-CRT). The place of CSP in the management of heart failure in the adult with the structurally normal heart continues to evolve. There is growing evidence that CSP may be superior to multisite pacing patients by some measures, but there is currently no randomized controlled trial demonstrating non-inferiority of CSP in terms of survival or other hard outcome. Larger randomized controlled trial and registry data are required to establish firmly the roles of multisite *versus* single site CRT. (Sharma and Vijayaraman, 2021; Vijayaraman et al., 2022).

The role of CSP for CRT in pediatric and CHD patients is just emerging but appears promising. A recent multicenter study of CSP in CHD patients compared conventional CRT to CSP in propensity score-matched subgroup of 25 patients. They demonstrated similar change in LVEF and complication rates, with a greater reduction in QRS duration with CSP (delta QRS duration 32 ± 29 ms vs 14 ± 25 ms; *p* = 0.03). (Moore et al., 2022). It is likely that the near future will bring further advances in our understanding of the role of CSP in CRT for pediatric and CHD.

### 2.4 Guidelines for CSP

Given the rapid evolution of CSP, guidelines are generally lagging behind clinical practice in non-CHD adults. The 2018 ACC/AHA/HRS bradycardia and conduction delay pacing guidelines endorse HBP specifically (or CRT) in patients with AV block and an indication for permanent pacing, especially if anticipated Vp>40% (Class 2A). (Kusumoto et al., 2019). The 2021 ESC guidelines on cardiac pacing and CRT again discuss only HBP, and not LBBAP. They place a Class 2A recommendation for consideration of HBP for those with failure of CS lead placement for CRT, and a Class 2B recommendation for consideration of HBP as an alternative to RV pacing in patients with AVB, LVEF >40% and anticipated Vp>20%. The role of HBP under other circumstances is not directly discussed, but there are clear and comprehensive summaries of available data in the supplementary tables. (Glikson et al., 2021). It is anticipated that the upcoming 2022 HRS/APHRS/LAHRS guidelines on physiological pacing, out for public review at time of writing, will be a significant step forward in establishing the role of CSP in the wider adult population.

For the pediatric and CHD population, the 2021 Pediatric CIED guidelines deliberately made no mention of CSP as recommendations for this patient group will also be included in the 2022 HRS/APHRS/LAHRS guidelines on physiological pacing. (Shah et al., 2021). For the management of ACHD patients, the 2014 North American arrhythmia guidelines pre-date almost all development of CSP, (Khairy et al., 2014) and HBP is mentioned only briefly and without specific recommendations in the 2018 EHRA/ESC/AEPC CHD arrhythmia guidelines (which are endorsed by HRS/PACES/APHRS and SOLAECE). (Hernandez-Madrid et al., 2018).

## 3 His Bundle Pacing

### 3.1 Implantation technique

The technique and equipment required for HBP in the adult with structurally normal heart is described in detail elsewhere. (Ravi et al., 2021). In general, the degree of technique modification required in pediatric/CHD patients depends upon the degree to which the anatomy varies from normal.

#### 3.1.1 Anatomy of the His-Purkinje system

The His Bundle (HB) is generally interpreted to be the part of the conduction system that lies between the AV node and bifurcating AV bundle (Figure 1). Some sub-bundles within the HB have been shown to be pre-destined to the LBB or the RBB, resulting in the correction of BBB in some patients following HBP. (Ravi et al., 2021). After exiting the common fibrous body, the HB passes through (or very close to) the membranous ventricular septum into the muscular septum, exiting on the ventricular crest before dividing to form the LBB and RBB. Variations in HB anatomy in the structurally normal heart have been well-described, with categorization into three types. Type I (47%) includes patients in whom the HB is covered in a thin layer of ventricular myocardium, Type II (32%) in whom the HB is deeper within the myocardium, and Type III (21%) in whom there are no myocardial surrounding fibers (“naked AV bundle”). (Nagarajan et al., 2019; Ravi et al., 2021). These variations result in different responses to decreasing pacing outputs: Type I typically transitions from NS-HBP at high output to S-HBP, Type II from NS-HBP to RVSP (without S-HBP at any output) and Type III from S-HBP to loss of capture.

In the pediatric patient with a structurally normal heart, the location of the proximal His Purkinje system is conventional, albeit that the smaller dimensions in children may be less amenable to lead delivery using standard sheaths such as the Medtronic (Medtronic Inc. Minneapolis, MN) C315H sheath (see below). However, the location of the His Purkinje system is clearly much more varied in patients with CHD. In simpler forms of CHD, such as an isolated VSD, the variation from normal is less marked, although the non-branching portion of the HB tends to be longer in those with perimembranous VSDs such as in tetralogy of Fallot. With increasingly complex CHD, the displacement of the AV node and HB becomes more marked. For example, in those with AVSD, the AV node is displaced postero-inferiorly, and in ccTGA (with situs solitus) the AV node is displaced anterosuperiorly with the long HB descending in the anterior portion (or “crest”) of the ventricular septum. (Anderson et al., 1983; Chubb et al., 2014).

#### 3.1.2 Equipment and implant

The majority of HBP in pediatric and CHD described in the literature has used Medtronic leads and sheaths. The 4.1Fr Medtronic 3830 has demonstrated good lead characteristics for implant with the narrow profile, catheter-based delivery system and fixed helix screw. In addition, there is longer-term lead robustness data available, including in the pediatric cohorts. (Shepherd et al., 2015). The lead is available in 49, 59, 69, and 74 cm lengths. Leads from Boston Scientific (Marlborough, MA, United States: Fineline II Sterox, 5.7Fr or Ingevity + lead, 6Fr) and Biotronik (Berlin, Germany: Solia S, 5.6Fr) have also been used successfully for HBP. (Ravi et al., 2021; O'Connor et al., 2022a).

The Medtronic C315H sheath is the most commonly used delivery sheath for HBP. It has a 7Fr outer diameter, 43 cm usable length and 79 mm/43 mm horizontal/vertical reach. It was designed for access from the left-sided device implant site to reach RA and RV septal locations, but can also be reshaped to a degree for right-sided implants, smaller patients or abnormal anatomy. (O'Connor et al., 2022a). Medtronic also produce deflectable delivery sheaths (with or without pre-shaping: C304His and C304, respectively), but they require a 9Fr introducer. Alternatively, coronary sinus delivery sheaths may be used for additional support, or alone, for more complex anatomies. (Ravi et al., 2021; O'Connor et al., 2022a). The use of the smaller Medtronic C315 S5 sheath in children has also been described. (Zizek et al., 2021).

Overall, in a recent multicenter study of CSP in CHD, 14 out of the 17 HSP were performed using the Medtronic C315H catheter, with only one utilizing the steerable C304H sheath. (Moore et al., 2022). Again, Boston Scientific (such as SSP3 sheath) and Biotronik (Selectra 3D) also produce appropriate sheaths, but these have been less widely employed in the pediatric and CHD cohorts. (O'Connor et al., 2022a).

Lead delivery is generally performed from the left side when venous access is available. However, in patients with a previous atrial switch operation and those with dextrocardia, some centers have deliberately chosen right‐sided venous access for better sheath orientation against the interventricular septum. (Cano et al., 2021).

#### 3.1.3 Identification of pacing site

HBP in pediatric and CHD patients is generally performed with the assistance of mapping tools. In a multicenter study including 17 CHD patients with HBP, 16 (94%) were performed using 3D mapping for guidance. (Moore et al., 2022). The application of electroanatomic mapping (EAM) techniques for HBP has been well described for both adults (Sharma et al., 2019) and pediatrics (Bury and Cortez, 2021). Alternatively, mapping using a steerable catheter, such as the Livewire octapolar catheter (Abbott Medical, Abbott Park, IL), and fluoroscopy alone has also been described. (Gordon et al., 2022).

For precise placement of the lead, mapping is performed in a unipolar fashion, with many choosing to display the signal on an electrophysiology recording system as well as at the pacing system analyzer. (Cano et al., 2021). When a His deflection is identified, generally aiming for ∼1:3 A:V ratio, pacing at 5V @1 ms identifies whether there is His bundle capture, with a faster sweep speed (eg 100mm/s) recommended for clarity. For those without a stable escape rhythm, pacemapping can be used to identify an adequate position based upon 12-lead morphology. (Cano et al., 2021). Placement of the lead typically requires 5–10 rotations, with subsequent careful assessment of parameters with the catheter withdrawn to the atrium.

A further consideration in pediatric implants is the degree of lead slack. Adult studies have noted increased rate of lead dislodgment or late rises in threshold with excessive HBP lead slack. (Ravi et al., 2021). Therefore careful evaluation of sufficient (but not excessive) lead slack is required in the growing patient.

#### 3.1.4 Identification of site of capture

**FIGURE 2:**
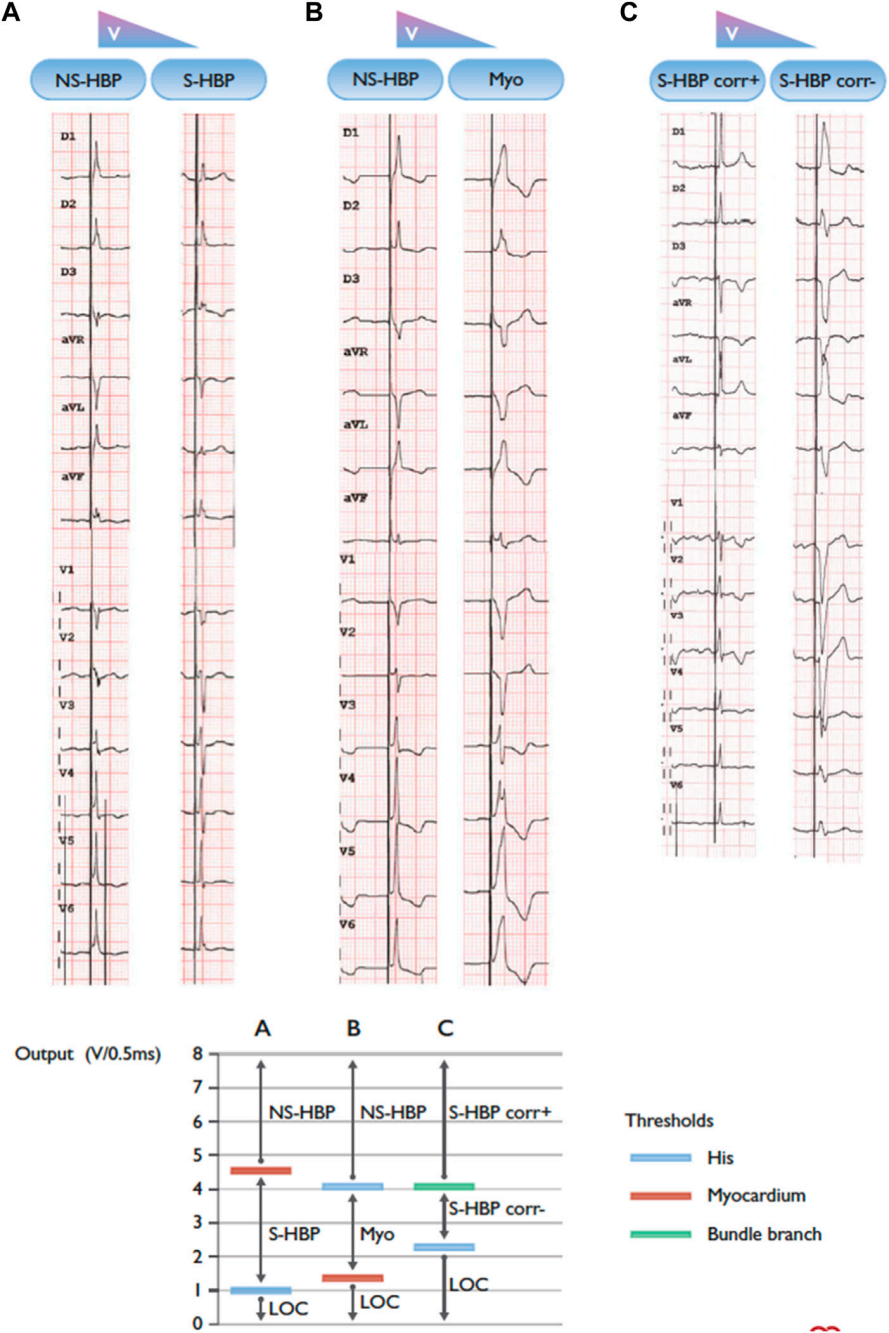
Three patients with different types of transitions in QRS morphology with His bundle pacing and decrementing pacing output. BBB = bundle branch block; Corr± = with/without correction of bundle branch block; LBBB = left bundle branch block; LOC = loss of capture; Myo = myocardium; NSHBP = non-selective His bundle pacing; S-HBP = selective His bundle pacing. **(A)** Non-selective to selective His capture. Note the presence of a “pseudo-delta” wave with non-selective capture and an isoelectric interval after the pacing spike with selective capture. **(B)** Non-selective His capture to myocardial capture only. **(C)** Selective His capture with correction of BBB to selective His capture with LBBB. Note: the graph on the right of the panel shows a schematic representation of the different thresholds in the three instances. Adapted from Glikson et al. (2021) with permission.

Identification of the type of HBP achieved is dependent upon recognition of the QRS morphology at varying outputs. Clear criteria for selective (S-HBP) *versus* non-selective (NS-HBP) have been defined, and it is reasonable to extrapolate those same criteria to pediatric and CHD patients. (Vijayaraman et al., 2018a). NS-HBP, with capture of the adjacent myocardium (Figure 1) is generally characterized by the presence of a pseudo-delta wave with near zero stim-QRS time (Figure 2). It should be noted, though, that the characterization of site of capture is more challenging in those with underlying bundle branch block which is resistant to proximal capture.

#### 3.1.5 Role of the backup RV lead

The evidence for the risks and benefits of a backup RV lead are well-delineated in the 2021 ESC pacing and CRT guidelines. (Glikson et al., 2021). A backup lead provides increased safety in the context of demonstrated rises in HBP lead thresholds, and can also assist programming, at the cost of increased system lead burden and complexity.

In the pediatric and CHD population, chronic rises in pacing lead thresholds have also been demonstrated and are generally in line with those reported in prior studies in non-CHD populations. (Vijayaraman et al., 2018b). Moore et al. (2022) multicenter study of CSP in CHD included 17 HSP patients, and there was average rise in HSP threshold from 0.8 ± 0.4V (at 0.5 ± 0.1 ms) to 1.6 ± 0.2V (at 0.5 ± 0.2 ms) at 1 year. Gordon et al. (2022) (23 HSP patients) identified a rise in ventricular capture threshold from average 0.7 ± 0.3V @0.4 ms at implant to 1.3 ± 0.5V @ 0.4 ms at median follow-up 610days (*p* <0.001). A backup RV lead was not reported to have been implanted in any case, and there were no severe complications related to loss of HSP capture in either study.

Similarly in congenital complete heart block (CCHB), there is generally a more robust underlying heart rhythm than in post-surgical iatrogenic CHB, and a backup RV lead has generally not been implanted in adults with CCHB. (Dandamudi et al., 2021).

### 3.2 Pediatric experience

The pediatric experience in HSP is summarized in Table 1, with the largest cohort reported by Gordon et al. (2022) with 17 pediatric HBP implants. Results have been generally encouraging with low complication rates. The majority of pediatric cases have been in larger (>40 kg) children, but a small number of sub-30 kg implants have been reported. (Zizek et al., 2021; Gordon et al., 2022).

**TABLE 1:** Summary of Conduction System Pacing (CSP) in publications with ≥5 pediatric and/or CHD patients (Kiehl et al., 2016).

Author	Number of patients				Patient mix					Indication		Prior device	Complications		Follow-up (months)
	*Total*	*His Bundle*	*LBBP*	*LVSP*	*Mean Age*	*Pediatric (<18 years)*	*CHD*	*Systemic LV*	*Baseline SVEF*	*AV block*	*Resynch-ronisation*		*His*	*LBBAP*	
Moore et al. (2022)*	65	17 (26%)	38 (58%)	10 (15%)	37 ± 21	17 (26%)	65 (100%)	65 (100%)	50 ± 14	54 (71%)	1 (2%)	28 (43%)	2 (4%)	1 (6%)	12
Cano et al. (2021)	20	10 (50%)	5 (25%)	5 (25%)	32 ± 17	5 (25%)	20 (100%)	13 (65%)	49 ± 14	17 (85%)	3 (15%)	11 (55%)	0	1 (5%)	16 (IQR 7–19)
Wenlong et al. (2022)	12	0 (0%)	10 (83%)	2 (17%)	8.2 ± 4.1	12 (100%)	4 (33%)	12 (100%)	65 ± 9	12 (100%)	0	0	0	0	12 (7–33)
Moore et al. (2020)	13^§^	11 (85%)	2 (15%)	0 (0%)	23 (IQR 15–36)	Unspecified	13 (100%)	0	10 mild	13 (100%)	0	7 (54%)	2^§^ (13%)		8 (IQR 3–14)
									3 mod						
									2 severe^¶^						
O'Connor et al. (2022a)	13	0	13 (100%)	0	55 ± 15	0	13 (100%)	11 (85%)	56 ± 8	7 (54%)	0	0	0	0	2
Gordon et al. (2022)	24	23 (96%)	1 (4%)	0 (0%)	14 (range 8–39)	17 (71%)	12 (50%)	1 (4%)	Unspecified	24 (100%)	0	6 (25%)	0	0	20 (range 8–25)
Dandamudi et al. (2021)	17	17 (100%)	0 (0%)	0 (0%)	27 ± 11	5 (30%)	0	17 (100%)	53 ± 14	17 (100%)	0	8 (47%)	2 (11%)	NA	19 ± 12

Note: * includes an unspecified number of patients also included in the publication by Gordon et al. (2022).

^a^ 2 out of total 15 attempted implants were unsuccessful.

^b^ Degree of systemic RV, dysfunction.

LBBP: left bundle branch pacing; LVSP: left ventricular septal pacing; CHD: congential heart disease.

Complications.

Moore et al. (2022)- wound dehiscence requiring reintervention at 1 month, significant increase in RV, pacing threshold and non-capture requiring reprogramming for 1 patient with HBP, and mechanical mitral valve endocarditis culminating in death in 1 patient 6 months postimplantation.

Cano et al. - ventricular pacing threshold rise requiring lead revision (LBBAP, lead).

Dandamundi et al. - two patients required lead revision during follow-up (at 722 and 14 days, respectively) due to significantly elevated HBP, thresholds.

There is also important and relevant data in a multicenter report of pacing of CCHB by Dandamudi et al. (2021) This study included 17 patients with CCHB (mean age 27 ± 11 years, 5 (30%) were <18 years). Eight had a history of prior pacing, including 3 with reduced LVEF. Reassuringly, an HB potential could be identified in every patient despite the presence of CCHB, and 4 (23%) received S-HBP with NS-HBP performed in the rest. Follow-up was documented at approximately 1 year, and they found an overall improvement in NYHA class (1.7 ± 0.9 *versus* 1.1 ± 0.3, *p* = 0.014). Notably, the SVEF in the PICM group improved from 26% ± 15% to 48% ± 3%.

However, two patients required revision of the HBP lead due to elevated pacing thresholds.

### 3.3 Adult congenital heart disease experience

The potential impact of surgical scarring near the conduction apparatus, and the variation in the underlying anatomy of the conduction system, have been raised as barriers to HBP in the CHD population. There is also a suggestion that those with CHD may be more prone to distal His Purkinje injury, and hence may not be able to achieve such a narrow QRS even with optimal lead placement. (Gordon et al., 2022).

The 2022 (non-ccTGA) multicenter study by Moore and others included 17 HBP systems in CHD patients, all of whom had biventricular circulation with systemic LV. TOF or VSD represented the commonest underlying CHD and in the majority the indication for pacing was AVN block, with only one implant for CRT alone. Nine (53%) systems were S-HBP, and 8 (47%) were NS-HBP. Again, there were no significant complications, but a rise of the ventricular pacing threshold to >2 V was documented in 3 (18%) patients. (Moore et al., 2022).

**FIGURE 3:**
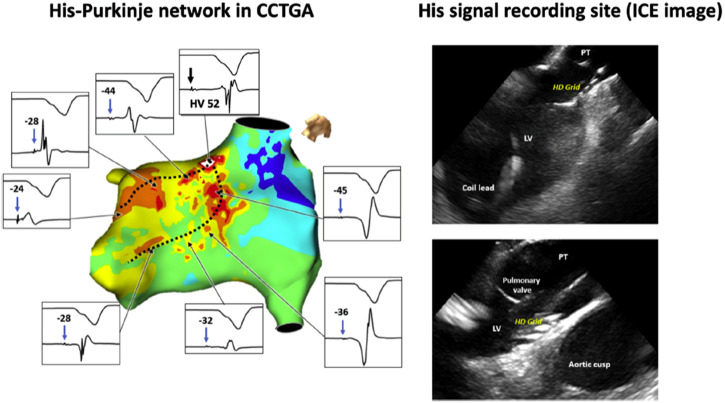
Left: Three-dimensional mapping of the conduction system in congenitally corrected transposition of the great arteries (CCTGA). Dashed black lines indicate the course of the morphologic left bundle branches. The septal aspect of the His bundle is located below the left ventricular outflow tract. Due to the anomalous His bundle course, the bundle enters the septum away from the mitral annulus. Right: Multipolar mapping catheter against the ventricular septum below the pulmonary valve annulus, where distal His-bundle electrograms were encountered. Intracardiac echocardiography (ICE) is used to demonstrate the position of the high-density multipolar catheter as it records His-bundle electrograms near the left ventricular outflow tract and pulmonary valve. LV = left ventricle; PT = pulmonary trunk. Reproduced with permission, Moore et al. (2020).

In a separate study of CSP in patients with uncorrected ccTGA, 11 out of 13 patients had HBP performed. The distal His bundle and proximal left bundle branches were identified within the morphologic left ventricle below the pulmonary valve, separate from the mitral annulus (Figure 3), and median HV at implant was 42 ms. At a median of 8 months follow-up, all patients were alive without significant change in pacing threshold or lead dysfunction. (Moore et al., 2020).

## 4 Left bundle branch area pacing

### 4.1 Implant technique

LBBAP is defined as the capture of either the proximal left bundle or one of its fascicles at a low threshold, usually also with septal myocardium capture at low output. (Huang et al., 2019). The technique and equipment required for LBBAP in the adult with structurally normal heart is described in detail elsewhere. (Ponnusamy and Vijayaraman, 2021). In general, the equipment employed is similar to that used in HBP, but the overall reported success rate is higher. Given the recent development of the technique, published experience in pediatric and CHD patients is limited, but the basic technique varies little from that in conventional hearts.

#### 4.1.1 Anatomy of the left bundle branch

The anatomical location of the LBB is closely tied to that of the distal HB, and similar considerations apply. (Anderson et al., 1983; Chubb et al., 2014). However, the LBB is much broader target with the fibers spanning the proximal subendocardial aspect of the left side of the interventricular septum, and therefore precise mapping (which would generally need to be performed from the systemic ventricle) has generally not been reported. The LBB itself typically has three main fascicles in the structurally normal heart, and the anatomical correlates of variations in this pattern across CHD morphologies has not been described in detail. (Anderson et al., 1983).

#### 4.1.2 Equipment and implant

Pre-implantation imaging should be reviewed to ascertain the thickness of the proximal septum and best estimate of location of the proximal LBB. The site of deployment of the LBBAP lead is generally performed empirically given the wider target for pacing. However, in some reported CHD cases, different techniques for site identification have been deployed. These include placement of a quadripolar mapping catheter across the HB to delineate the distal extent of His electrograms (Ponnusamy and Vijayaraman, 2021), RV angiography (20 (42%) of the cases reported by Moore et al. (2022) (Moore et al., 2022)), or EAM. O'Connor et al. (2022a) (13 ACHD LBBAP subjects) used EAM in those with more complex anatomy, such as atrial switch for d-TGA, while Moore et al. (2022) multicenter study reported use of EAM in 3 (6%) cases.

Pacing system equipment for LBBAP is similar to that employed for HBP. In Moore et al. (2022) multicenter study, 31 out of 48 LBBAP were performed using the C315H catheter (65%), with a variety of other catheters used for the remaining cases (including CS delivery system or C304H steerable sheath). O'Connor et al. (2022b) reported that 7 out of 13 CHD LBBAP cases required reshaping of the C315H sheath. Sheath reshaping should be performed with the dilator in place to avoid sheath kinking. For those with severely dilated atria, use of the C315H sheath inside a multi-purpose CS guide sheath (with the proximal 12 cm cut off, including the hemostatic hub) was reported. (O'Connor et al., 2022a).

#### 4.1.3 Guidance of deployment

The successful LBBAP site is classically reported to be 1–1.5 cm below the distal HB, along a line connecting the HB to the RV apex in RAO view. More recent data suggests that many operators are achieving capture at slightly more distal sites (i.e., left bundle fascicular pacing, rather than common LBB), 1.5–4.5 cm distal to the His. (Jastrzebski et al., 2022a). In the structurally normal heart, the successful location typically demonstrates a “W” pattern in lead V1 on pacing, with tall R wave in lead II, RS in lead III and discordant QRS complexes in aVR and aVL. (Ponnusamy and Vijayaraman, 2021). Paced ECG correlates of successful implant sites have not been established in more complex CHD patients.

Once the site for deployment is selected, there are two main methods of guidance of lead delivery: gradual screwing-in of the lead with monitoring of paced QRS morphology and unipolar impedance, or rapid screwing-in with monitoring of PVC morphology. All data regarding these techniques is isolated to adults with structurally normal hearts. (Ponnusamy and Vijayaraman, 2021). However, in the smaller pediatric patients, additional guidance with measurement of depth within septum using contrast or transesophageal echocardiography may also prove important. (Huang et al., 2019; Moore et al., 2022; Wenlong et al., 2022). In the context of a thinner interventricular septum, it should be noted that the distal edge of the ring electrode on the 3830 lies approximately 10 mm from the tip of the helix, and the proximal edge of the radiopaque marker lies just under 18 mm from the helix tip (helix ∼1.8 mm, helix origin to distal aspect of the ring electrode ∼9 mm, ring electrode ∼4 mm, radiopaque marker proximal to ring electrode ∼3 mm). Contrast may be injected through the delivery system, delineating the margins of the septum and hence assisting estimation of the implant depth.

#### 4.1.4 Types of capture

Multiple forms of LBBAP capture are recognized, and the main types are summarized in Figure 1. In initial work on LBBAP, no firm criteria for the different forms of LBBAP had been established, but the work of Wu et al. (2021), using a multipolar LV septal catheter and additional HBP lead at implant, has provided greater clarity of the mechanisms and recognition of S-LBBP, NS-LBBP and LVSP. In brief, a range of criteria were defined (Table 2) and in clinical practice a combination of criteria are used to confirm LBBAP. The shorter effective refractory period of LV myocardium (*versus* LBB) may also be used to prove LBB capture, using either a basic drivetrain or sensed extras. (Jastrzebski et al., 2020).

**TABLE 2:** Electrophysiological Parameters and Values for confirming LBB capture. LBB: left bundle branch; LBBP: left bundle branch pacing; NS: non-selective; RBBB: right bundle branch block; Stim-LVAT: stimulus to left ventricular activation time; Stim-QRSend: time from stimulus to the end of QRS. Adapted from Wu et al. (2021).

Electrophysiological parameters	Electrophysiological definition	Value for confirmation of LBB capture
Paced RBBB pattern	qR and rsR, without terminal s in lead V_1_	Has a sensitivity of 100%
		Necessary but not sufficient
LBB potential	High-frequency signal at LBB lead with potential to ventricular interval: 20–30 ms	Indicates the lead is at or near the LBB
		A good indication for LBB capture, but not a direct sign of LBB capture
QRS duration	From the onset of QRS to the end of QRS during selective LBBP	Selective capture may result in a longer paced QRS duration than that in NS capture
	From the end of the stimulus artifact to the end of QRS during NS-LBBP and LVSP	Paced QRS duration was not helpful to distinguish LVSP from LBB capture
Stim-QRS_end_	From the onset of the stimulus artifact to the end of QRS	Selective capture may result in a longer Stim-QRS_end_ than that in NS capture
Stim-LVAT	From the onset of the stimulus artifact to peak R-wave in lead V6	Abrupt shortening of Stim- LVAT of ≥10 ms during increasing output (specificity: 100%)
		Stim-LVAT ≤75 ms in patients with non-LBBB (specificity: 95%; sensitivity 82%); Stim- LVAT ≤85 ms in LBBB (specificity: 93%, sensitivity 76%)
Selective LBBP	Discrete component and isoelectric interval on intracardiac electrogram	Has a specificity of 100%
	M or rsR′ and wide R′ with a notch in lead V1	
Electrophysiological Criteria requiring additional leads or catheters		
Retrograde His potential	His potential recorded from HBP lead during LBBP lead pacing	Direct evidence for confirmation of LBB capture
Anterograde left conduction system potential	Potential recorded from multipolar LV septal catheter during LBBP lead pacing or intrinsic rhythm	Direct evidence for confirmation of LBB capture

It should be noted that, in contrast to HBP, the QRSd is longer with S-LBBP, as the local myocardial capture serves to reduce the degree of RBBB. However, the time to activate the lateral wall of the LV should remain constant in both S-LBBP and NS-LBBP as it is not dependent upon the capture of local myocardium. This activation time is identified as the time from stimulus to peak activation in V6, variably termed “LV Activation Time” (LVAT) or “R Wave Peak Time” (RWPT): the former term is more widely employed, while the latter is arguably more physiologically accurate. In adults without LBBB, an LVAT≤75 ms is both sensitive and specific for LBB capture (≤85 ms in LBBB). Acceptable values in pediatric and CHD have not been established, but appear to be slightly shorter in children. In a study including 12 children with LBBAP, Wenlong et al. (2022) found an LVAT of 61 ms (IQR 59–63), similar at 1.5V, 3V, and 5V. In CHD patients with LBBAP, Moore et al. (2022) found an average LVAT of 73 ± 19 ms, and O'Connor et al. (2022a) 66 ± 6 ms. The V6-V1 interval has also been suggested more recently as a highly specific marker of LBBAP, using the cutoff of >44 ms. (Jastrzebski et al., 2022b). The cutoff is likely similar in CHD, with Moore et al. (2022) identifying an average V6-V1 interval of 47 ± 17 ms in those with LBBAP. (Moore et al., 2022).

In CHD patients, it is likely that identification of the LBB may prove more challenging as the field evolves to include ever more complex patients. In this setting, LVSP may often be achieved, and it remains to be proven that this is significantly worse in terms of hard outcomes in comparison to LBB pacing. LVSP is typically defined as when LBB capture criteria are not met, but a deep septal position may be visualized with contrast and there is a significant reduction in the QRSd (or QRS≤130 ms). (Zhu et al., 2022).

### 4.2 Pediatric experience

The largest published study of pediatric LBBAP is by Wenlong et al. (2022), with the majority of remaining experience limited to isolated case reports, or cases within larger cohorts of CSP. (Ponnusamy et al., 2020; Gordon et al., 2022). Wenlong et al. (2022) reported 12 pediatric cases (Table 1), with maximum follow-up of 330 months. Minimum weight was 130 kg, with minimum intraventricular septal thickness 50 mm, and no procedure-related complications were reported. The method used for identifying the correct depth of septal lead penetration was based upon paced morphology and impedance, augmented by depth visualization using contrast injected *via* the C315 S4 or HIS sheath in the LAO view.

### 4.3 Adult congenital heart disease experience

There is growing experience of LBBAP in CHD. Moore et al. (2022) multicenter study, included 48 LBBAP, (2 selective, 36 non-selective, 10 LV septal), and O’Connor et al. reported 13 CHD LBBAP. Results have been generally encouraging, with low complication rates (Table 1).

One particular area of interest relates to the application of LBBAP to patients with systemic right ventricles. There are two main groups: those without operative anatomic correction of ccTGA (who are also at increased risk for AVB and therefore represent a disproportionately high percentage of ventricularly paced ACHD patients) and those with d-TGA post atrial switch. In the ccTGA patient, there remain concerns regarding both the efficacy and safety of pacing the superficial and relatively exposed LBB. LBBAP results in continued delayed transseptal activation, and the long-term viability of the vulnerable His Purkinje system has not been proven. Therefore, RBB area pacing has been proposed (burrowing across the septum to reach the RBB on the left side) and performed with good initial results in a small subset of three patients. (Namboodiri et al., 2022). Similarly, superficial LBBAP has been performed in patients with d-TGA and atrial switch, but questions remain as to whether achieving RBB area pacing (again burrowing across septum to achieve early activation of the bundle associated with the sub-systemic ventricle) might be more beneficial, albeit more challenging. (O'Connor et al., 2022a).

## 5 Risks, complications and future directions

### 5.1 Selection of form of CSP

CSP is a relatively new technique, and the data on which to base the decision as to whether to perform CSP in pediatric/CHD is limited. The data is even more limited for LBBAP, and therefore decisions for CSP modality are likely to evolve rapidly.

HSP is backed by the greater evidence for short term efficacy and safety, has a lower risk of perforation, and better data on feasibility of extraction (see below). However, there remain concerns regarding lead longevity and reliability, particularly in the context of implants for AVB in the majority of the pediatric/CHD population. Recent data from a single study of CSP following AV nodal ablation (in the structurally normal adult heart) demonstrated reassuringly similar safety outcomes in both HBP and LBBAP groups, but with inferior lead parameters in the HBP cohort. (Pillai et al., 2022).

Perhaps the greatest determinant of CSP modality in the long term in pediatric and CHD will be the difference in learning curves. In contrast to adult electrophysiology, even the largest pediatric and CHD centers rarely perform over 50 device implants per year. It has been suggested that approximately 40 implants are required for the learning curve to begin to plateau for HBP, with substantially smaller number of implants required to learn LBBAP techniques. (Kircanski et al., 2022).

#### 5.1.1 Long-term lead trends

**FIGURE 4:**
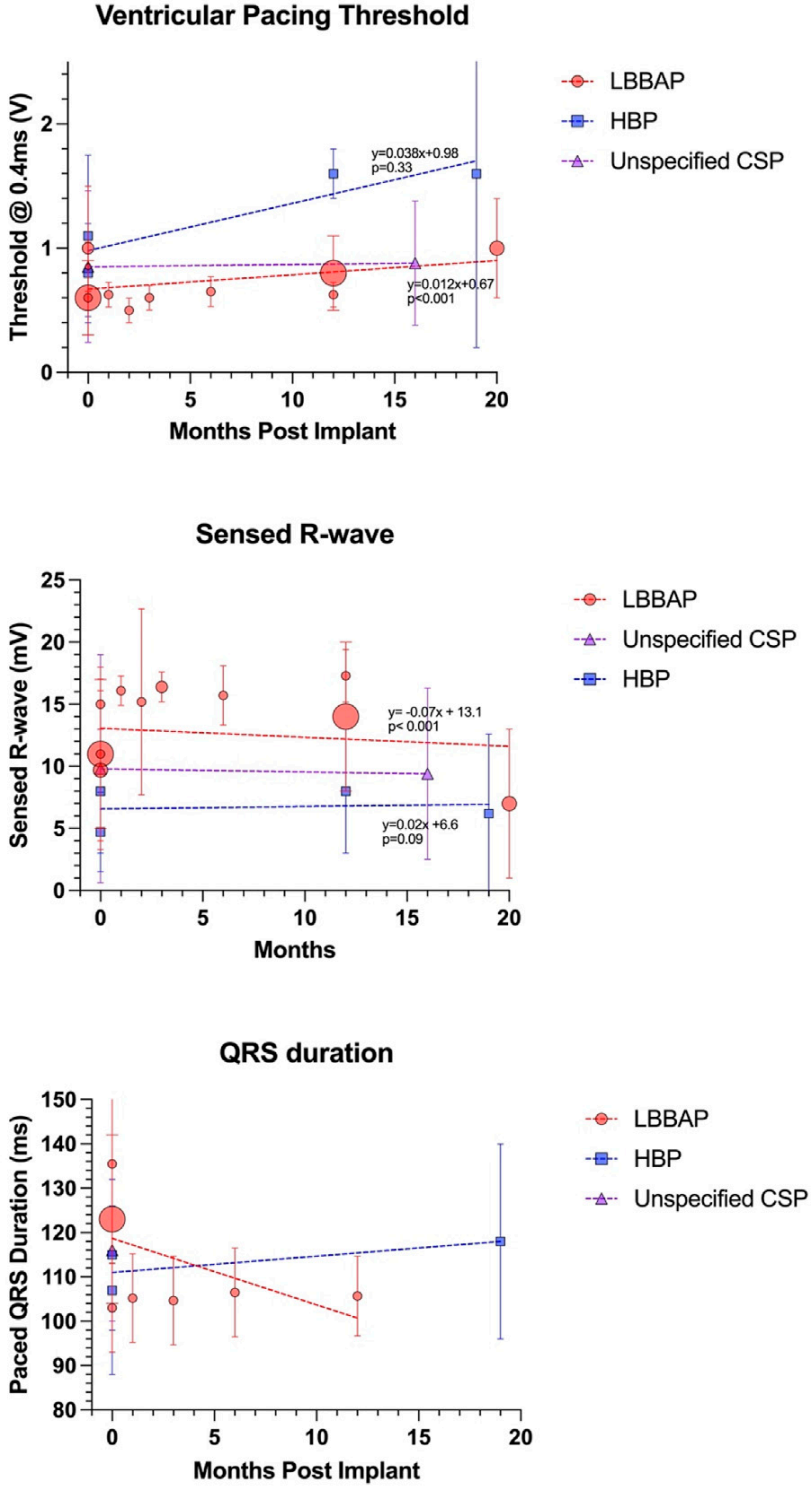
Long term lead trends as reported by the studies outlined in Table 1. Size of data point is proportional to number of subjects. Regression equations and *p*-values reported for LBBAP and HBP only (note that Cano et al. did not separate HSP *versus* LBBAP lead parameters).

In general, the reported rise in thresholds over time, and the fall in sensed R-wave, has been demonstrated to be greater for HSP *versus* LBBAP in the adult population. (Moore et al., 2022). Interestingly, this is not necessarily observed when reviewing the (limited) aggregate pediatric and CHD data, summarized in Figure 4. Detailed data for longitudinal trends in HBP are available for fewer patients and therefore the *p*-values should be interpreted with caution, but current data does not currently demonstrate any inferiority of HBP long-term lead trends compared to LBBAP in pediatric and CHD patients.

#### 5.1.2 Lead extraction

Lead extraction of CSP leads remains a significant concern, particularly in younger patients where multiple leads are anticipated over a lifetime. The Medtronic 3830 lead extraction data is currently based in large part on the experience in pediatrics, where it has proven to be more easily extracted than conventional leads. (Shepherd et al., 2015). Extraction of the less deeply implanted HBP lead has also been demonstrated to be feasible in ever larger cohorts, with subsequent successful re-implant of new HBP lead in many cases. (Wijesuriya et al., 2022).

The extraction of the more deeply implanted LBBAP lead is of greater concern, and experience is currently extremely limited. There are reported case reports of lead extraction but leads have all been <2 years old. (Vijayaraman, 2020; Wijesuriya et al., 2022). There are also concerns of potential weakness at the stress point of the fulcrum of the lead as it enters the septum: the lead was not specifically designed for such a deep implant. Reports of stress fractures are rare, but partial extraction may prove to be more common. (Chen et al., 2021).

#### 5.1.3 Device programming

Challenges in the programming of HBP systems have been well documented, but there is no specific work aimed at the pediatric or CHD populations. (Ravi et al., 2021). Many will program the HBP output at high amplitude (eg 5V @1 ms) regardless of threshold for at least 3 months, and automatic capture threshold algorithms should generally be turned off. Particular care should also be taken in tailoring the sensitivity based on A and V amplitudes to avoid over- and/or under-sensing, and the programmed delay is generally 30–50 ms shorter than conventional pacing to take account of the HV delay. HOT-CRT or back-up RV pacing present further complexity for programming but increased safety. LBBAP requires minimal adaptations, but consideration of a slightly shorter AV delay should also be given. (Ponnusamy and Vijayaraman, 2021).

### 5.2 Future directions

A recent survey of 185 adult electrophysiologists found that the majority (85%) of respondents anticipated that CSP would dominate bradycardia pacing in the future. (Kircanski et al., 2022). No such poll of pediatric or CHD implanters has been performed, but it is likely that the field will follow in the same direction as evidence for long term efficacy and safety accumulates. The use of CSP for CRT is also attractive for many scenarios. The same adult survey suggested that 72% believed that CSP would dominate CRT in the future. In this context it is interesting that Moore et al. (2022) identified that CSP resulted in greater QRS narrowing that CRT, and at least non-inferior improvement in LVEF, on comparison with a propensity score matched CRT cohort.

HOT-CRT and LBBAP-optimized CRT clearly may also play a role in those with more conventional cardiac arrangements and CS anatomy. However, the greatest challenges, and gains, in implementation of CSP for CRT arises in the more complex anatomies or physiologies. In particular, minimization of dyssynchrony of the single ventricle is increasingly recognized to be important for long-term survival. (Chubb et al., 2022). Future work may demonstrate that recruitment of the conduction system is worth the risk of a ventricular lead in the systemic circulation in selected cases. Alternatively, leadless systems such as the Wise-CRT implant (EBR Systems, Sunnyvale, CA, United States) may also provide substantial benefit on capture of the LBB, and research in that area is on-going. (Elliott et al., 2022).

Finally, dyssynchrony of the right ventricle has also been recognized to have substantial impact upon the function of both ventricles, such as in ToF. Permanent HBP in patients with RBBB has been demonstrated to be associated with significant narrowing in the QRSd and improvement of LV function, and therefore this area warrants further exploration. (Sharma et al., 2018). It is likely that the surgical transection of branches of the RBB will prove more resistant to proximal re-recruitment. Recent data, though, has demonstrated that the more distal right sided conduction system is relatively intact. (Verzaal et al., 2021).

## 6 Conclusion

The adoption of conduction system pacing for adults with structurally normal hearts has evolved rapidly over the past 5 years, with growing evidence of safety and improved outcomes in comparison to conventional pacing modalities. For children and patients with congenital heart disease, the potential benefits of CSP are even more substantial, but this must be weighed against increased procedural complexity and possible increased risk in these patient cohorts. Most recently, there has been a shift from His bundle pacing towards left bundle branch area pacing, and pediatric/CHD-specific publications are now creating a greater evidence base for CSP. Continued work is required to delineate the role that CSP has to play in the future, but work in the structurally normal heart suggests that the use of CSP in pediatrics and CHD is likely to grow rapidly.
